# Effect of Solution Temperature on the Microstructure and Properties of AlSi37Cu0.7Mg0.9Ni0.2 Alloy Prepared by Rapid Solidification and Hot Extrusion

**DOI:** 10.3390/ma18225244

**Published:** 2025-11-20

**Authors:** Xiaodong Mao, Zhenning Chen, Ningjie Gu, Dongnan Huang, Linzhong Zhuang

**Affiliations:** 1 State Key Laboratory for Advanced Metals and Materials, University of Science and Technology Beijing, Beijing 100083, China; 2 Beijing Advanced Innovation Center for Materials Genome Engineering, University of Science and Technology Beijing, Beijing 100083, China; 3 Chinalco Material Application Research Institute Co., Ltd., Beijing 102209, China

**Keywords:** AlSi37Cu0.7Mg0.9Ni0.2 alloy, solution temperature, anodizing, mechanical properties

## Abstract

This study systematically investigated the effects of solution temperature (460–560 °C) on the microstructure, mechanical properties, and corrosion behavior of AlSi37Cu0.7Mg0.9Ni0.2 alloy rods prepared by rapid solidification and hot-extrusion. The results demonstrated that the solution temperature critically governed the alloy’s recrystallization behavior, precipitation kinetics, and phase distribution. With the increase in solution temperature, the alloy exhibited progressive grain coarsening (from 4.51 μm at 460 °C to 13.25 μm at 560 °C) and enhanced precipitation hardening, leading to a 108.8% increase in hardness (198.4 HV at 560 °C) but a concurrent reduction in ductility (from 2.5% to 1.0%). Electrical conductivity initially improved by 3.4% at 460 °C (27.44% IACS) compared with the extruded state, but deteriorated at higher temperatures due to increased electron scattering. Anodic oxidation tests revealed a non-monotonic corrosion trend, with maximum weight loss (57.50 mg·g^−1^) occurring at 480 °C due to microstructural inhomogeneity, while higher temperatures (560 °C) partially restored corrosion resistance. Electrochemical analysis corroborated these findings, showing the 480-treated sample exhibited the lowest corrosion potential (−1.0159 V). Microstructural characterization confirmed that optimal mechanical properties were achieved through a combination of fine *β*″-Mg_2_Si (<20 nm), *θ*′-Al_2_Cu precipitates, and thermally stable Al_3_Ni phase. These results established a comprehensive process-structure-property relationship, which provided critical guidance for tailoring the alloy’s performance in structural–functional applications.

## 1. Introduction

As a transformative innovation in advanced materials processing, rapid solidification technology (RST) [1,2,3,4,5] achieves extraordinary cooling rates (typically 10^3^–10^7^ K/s) that enable non-equilibrium solidification of metallic melts. In stark contrast to conventional casting processes (with cooling rates of only 10^−1^–10^−2^ K/s), RST facilitates remarkable microstructural refinement, substantial extension of solid solubility limits, and generation of metastable phases, collectively leading to dramatic improvements in critical material characteristics, including mechanical properties, thermal stability, and corrosion resistance. Among various RST approaches, melt spinning [6,7,8,9,10,11] has emerged as particularly noteworthy due to its exceptional processing capabilities (achieving cooling rates up to 10^5^–10^7^ K/s). This versatile technique has found extensive applications in producing cutting-edge microcrystalline/nanocrystalline materials, bulk metallic glasses, and advanced functional magnetic materials.

High-silicon aluminum alloys (Al-Si alloys with 12–50 wt.% Si content) [12,13,14,15,16,17,18,19] have emerged as a prominent class of lightweight structural–functional-integrated materials, distinguished by their exceptional combination of performance characteristics. These alloys simultaneously offer the structural advantages of low density, high specific strength, and elevated elastic modulus, while demonstrating remarkable functional properties including outstanding wear resistance, intrinsic self-lubricating capability, excellent thermal conductivity, and an exceptionally low coefficient of thermal expansion. These alloys demonstrate tremendous potential for high-end applications, particularly in aerospace structural components, automotive lightweight systems, and advanced electronic packaging. Nevertheless, conventional manufacturing methods frequently fail to satisfy the stringent performance demands of high-silicon aluminum alloys, making rapid solidification technology an essential fabrication approach for these materials. Notably, previous studies have demonstrated that AlSi37Cu0.7Mg0.9Ni0.2 alloy rods fabricated through melt spinning–hot extrusion exhibit exceptional performance in anodizing applications. This alloy serves as a critical interfacial material in current transmission systems, effectively bridging titanium alloy hangers and aluminum alloy workpieces that function as conductive electrodes. The synergistic enhancement of its mechanical properties, electrical conductivity, and corrosion resistance plays a pivotal role in determining the overall reliability and performance of the system.

The rapidly solidified and hot-extruded AlSi37Cu0.7Mg0.9Ni0.2 alloy undergoes significant microstructural evolution through heat treatment. Solution treatment facilitates the complete dissolution of alloying elements (Si, Cu, Mg, and Ni) into the *α*-Al matrix, creating a highly supersaturated solid solution. Subsequent artificial aging treatment induces controlled precipitation of secondary phases from this metastable state, enabling precise tuning of the alloy’s mechanical properties and corrosion resistance [20,21,22,23]. The solution temperature represents the most critical parameter in the solution treatment process. At constant holding durations, insufficient solution temperatures result in incomplete re-dissolution of alloying elements, which subsequently compromises the precipitation strengthening efficacy during aging. Conversely, excessive solution temperatures induce grain coarsening and may cause microstructural overheating or even localized melting, leading to severe degradation of mechanical properties. For the AlSi37Cu0.7Mg0.9Ni0.2 alloy system, the high concentration and diversity of alloying elements, coupled with intricate secondary-phase strengthening mechanisms, render the solution heat treatment parameters particularly critical for controlling the final microstructure and optimizing performance characteristics. Therefore, this study systematically investigated the effect of solution temperature on the microstructural evolution, mechanical performance, and corrosion behaviors of rapidly solidified and hot-extruded AlSi37Cu0.7Mg0.9Ni0.2 alloy rods. The aim of this paper is to establish a comprehensive understanding of the solution temperature-dependent microstructural characteristics and their correlations with performance metrics. Furthermore, the underlying mechanism governing surface conductivity during anodic oxidation will be clarified. These findings will represent a substantial advancement in establishing fundamental process–structure-property relationships, providing crucial insights for optimizing the alloy’s anodization performance.

## 2. Experimental Materials and Methods

**Figure 1:**
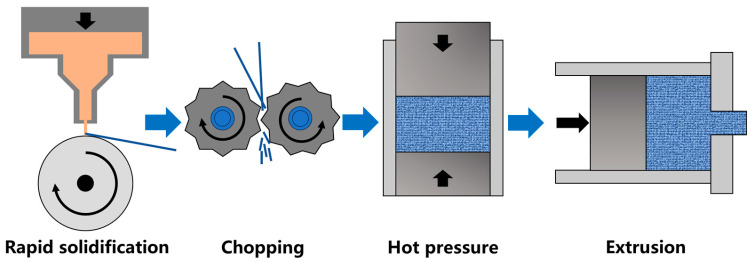
Schematic diagram of preparation process for AlSi37Cu0.7Mg0.9Ni0.2 alloy rods [24].

The test material was an AlSi37Cu0.7Mg0.9Ni0.2 alloy rod prepared by rapid solidification–vacuum hot pressing–hot extrusion deformation. The preparation process was illustrated in Figure 1, and the composition of the alloy was shown in Table 1. The initial diameter of the hot-pressed billet was Φ 80.0 mm, and the size of the finished bar was Φ 16.0 mm. The heating temperature of the hot-pressed billet was set at 450–460 °C, the extrusion outlet temperature was 380–390 °C, the extrusion speed was 0.2–0.4 mm/min, and the extrusion ratio was 25. The extruded state (ES) rod samples were placed in the Thermconcept-3508 air heating furnace for solution treatment. The temperatures were set at 460 °C, 480 °C, 500 °C, 520 °C, 540 °C, and 560 °C, holding for 2 h, followed by quenching in room-temperature water (transfer time < 15 s). After quenching, artificial aging was performed at 180 °C for 8 h immediately.

Mechanical properties were tested on the alloys in different states. The tensile specimens were prepared in accordance with GB/T 228.1-2021 [25]. The tensile direction was aligned with the hot extrusion direction, and the tensile speed was set at 2 mm/min. For each state, three parallel specimens were tested, and the average value was calculated. Vickers hardness measurements were performed on the aforementioned specimens using a VH-5LAC hardness tester (Matsuzawa Co., Ltd., Sayama, Japan) under a 49 N load with a 15 s dwell time. Each specimen was measured at eight different locations, and the average value was recorded. The electrical conductivity of the AlSi37Cu0.7Mg0.9Ni0.2 alloy rods in different states was measured on cross-sections using a SIGMATEST-2 eddy current conductivity meter (Helmut Fischer Gmbh, Sindelfingen, Germany). Five measurements were taken at different positions for every state, and the average values were determined.

Anodic oxidation corrosion weight loss tests were performed on the alloy samples above with a size of Φ 15 mm × 20 mm. The testing procedures were as follows: (1) alkaline washing: wash the alloy samples with 10% NaOH solution for 15 s at 55–60 °C; (2) water washing: wash with deionized water for 15 s; (3) acid washing: wash with 30% HNO_3_ solution for 30 s; (4) water washing: wash with deionized water for 15 s; (5) anodic oxidation: sulfuric acid solution 240 g/L, oxidation voltage of 12V, oxidation time of 65 min; (6) water washing: wash with ultrasonic deionized water for about 200 s; (7) drying: dry the samples at 100 °C for 10 min; (8) weigh and record the weight of the alloy samples before and after every cycle. The corrosion weight loss curves of different samples were plotted after 10 cycles.

**Figure 2:**
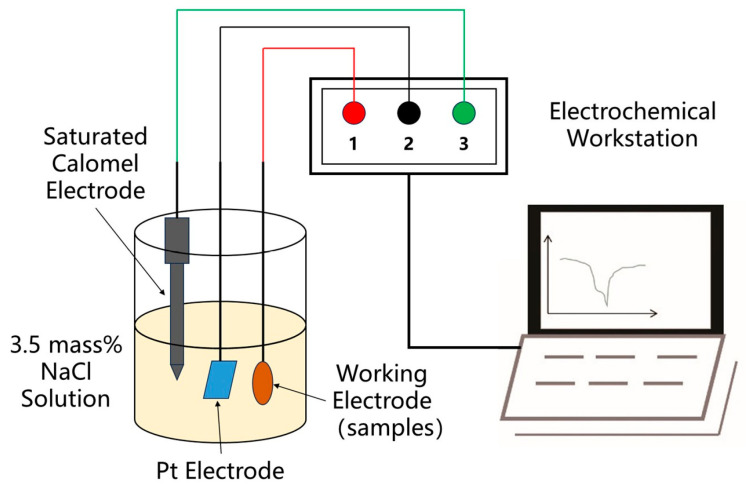
Principle of electrochemical working station.

Polarization curve tests of the alloy samples above were performed on the PGSTAT302N electrochemical workstation, and the sample size was set as Φ 15 mm × 5 mm. The test surfaces were ground, polished, cleaned, and dried. The three-electrode system was adopted in the experiment, i.e., the alloy sample as the working electrode, the platinum plate as the auxiliary electrode, and the saturated calomel electrode as the reference electrode. The 3.5 mass% NaCl solution was set as the test solution. The potential scanning rate was set as 1 mV/s, and the scanning range was set as −1.2~−0.2 V. The principle of the electrochemical working station is shown in Figure 2.

The metallographic microstructure of the alloys was observed using an Axio-Lab A1 metallographic microscope (Carl Zeiss AG, Jena, Germany). After grinding and polishing, the samples were corroded using Keller’s reagent for 30 s. Then the alloy samples were analyzed by electron backscatter diffraction (EBSD) using a TESCAN MIR3 field emission scanning electron microscope (TESCAN GROUP, a.s., Brno, Czech Republic). The size of the test step was set to 0.5 μm, and the size of the scanning image was set to 300 μm × 250 μm. The angle difference in grain boundaries and the recrystallization degree of the alloys were statistically characterized.

To observe the morphology of precipitates during aging in different alloy samples, the Empyream X-ray diffractometer (XRD) (Malvern Panalytical, Almelo, The Netherlands) was used to analyze the phases of alloys in different states using CuKα radiation lamp (*λ* = 0.154056 nm). The scanning rate was set at 2°/min with a step size of 0.02°, and *2θ* was continuously scanned in the range of 20~90°. JEM-F200 transmission electron microscopy (TEM) (JEOL Ltd., Tokyo, Japan) was used to observe the precipitates. The samples were prepared on an ion thinning instrument with an acceleration voltage of 200 kV, and the composition of precipitates was tested using an energy dispersive spectrometer (EDS).

## 3. Results and Discussion

### 3.1. Mechanical and Electrical Conductivity Properties

**Figure 3:**
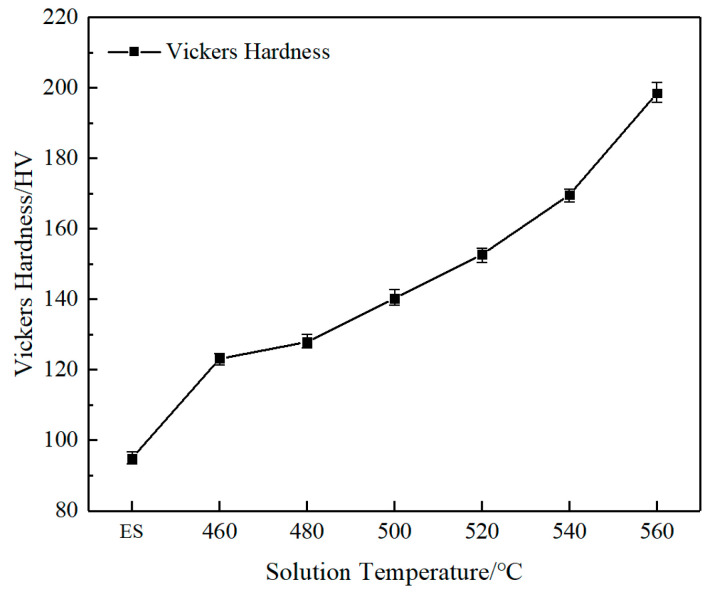
Vickers hardness of AlSi37Cu0.7Mg0.9Ni0.2 alloy under different states.

The Vickers hardness curves of AlSi37Cu0.7Mg0.9Ni0.2 alloy under extruded state and different solution temperatures are shown in Figure 3. It can be seen that the Vickers hardness of the extruded alloy rod was 95.0 HV, which was relatively low. The curve showed that the hardness of the alloy displayed a significant increase after T6 heat treatment. The hardness reached 123.2 HV at the solution temperature of 460 °C, with an increase of 29.7% compared to the extruded state. As the solution temperature rose further, the hardness continued to increase. The hardness of AlSi37Cu0.7Mg0.9Ni0.2 alloy reached the top at 560 °C, and the value was 198.4 HV, with an increase of 108.8%.

**Figure 4:**
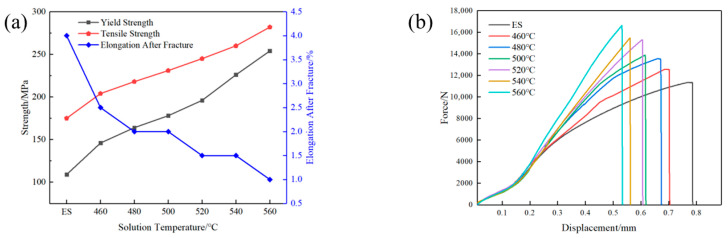
Mechanical properties of AlSi37Cu0.7Mg0.9Ni0.2 alloy under different states. (**a**) Mechanical properties; (**b**) force–displacement curves.

Figure 4 shows the variation curves of tensile mechanical properties of AlSi37Cu0.7Mg0.9Ni0.2 alloy under different states. It can be seen that the yield strength, tensile strength, and elongation after fracture of the extruded state alloy rod were 109 MPa, 175 MPa, and 4.0%, respectively, which indicated that the extruded alloy had lower strength and higher plasticity. It can be seen that the yield strength and tensile strength of the extruded alloy bars gradually increased with the rise in solution temperature, from 156 MPa and 204 MPa at 460 °C to 254 MPa and 282 MPa at 560 °C after T6 heat treatment. However, the elongation after fracture gradually decreased from 2.5% at 460 °C to 1.0% at 560 °C. The experimental results demonstrated that the increase in solution temperature effectively enhanced the strength of AlSi37Cu0.7Mg0.9Ni0.2 alloy while simultaneously deteriorating its plasticity. This inverse strength-plasticity relationship clearly revealed the crucial role of solution temperature in governing the alloy’s mechanical properties through microstructural modifications. The progressive increase in solution temperature enhanced the dissolution of strengthening phases within the matrix of the alloy, which promoted the formation of a higher density of finer and more uniformly distributed precipitates during subsequent aging treatment. This microstructural refinement mechanism contributed substantially to the strength enhancement through optimized precipitation hardening effects. However, microstructure evolution, such as grain growth and second-phase coarsening, led to a sustained decrease in plasticity at the same time.

**Figure 5:**
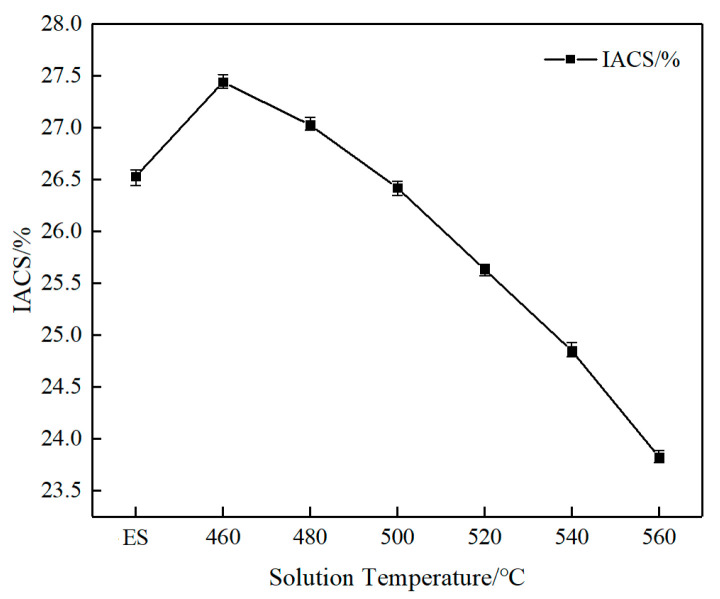
Conductivity of AlSi37Cu0.7Mg0.9Ni0.2 alloy under different states.

Figure 5 shows the variation curve of electrical conductivity for AlSi37Cu0.7Mg0.9Ni0.2 alloy rods under different heat treatment conditions. In the extruded state, the alloy demonstrated a conductivity of 26.53% IACS. Notable changes in conductivity were observed, followed by the solution and aging treatments. The optimal conductivity of 27.44% IACS was achieved at the solution temperature of 460 °C, representing a 3.4% improvement over the extruded state. However, further increase in solution temperature resulted in a progressive decline in conductivity, with values decreasing to 23.82% IACS at 560 °C, corresponding to a 13.19% reduction relative to the 460 °C condition and a 10.2% decrease compared to the extruded state. These findings reveal a dual effect of solution temperature: at lower temperatures (460 °C), the conductivity surpasses that of the extruded alloy due to favorable precipitation and optimized distribution of solute atoms. While the solution temperature exceeded 500 °C, the conductivity of the alloy declined below the extruded state, which was primarily attributed to grain coarsening and dissolution/redistribution of second phases that enhance electron scattering. The observed non-monotonic temperature dependence underscores the importance of precise thermal processing control for optimizing the electrical performance of AlSi37Cu0.7Mg0.9Ni0.2 alloy.

### 3.2. Anodic Oxidation Corrosion and Electrochemical Performance

**Figure 6:**
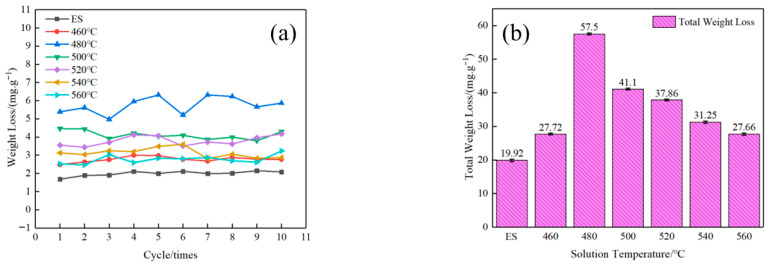
Anodizing corrosion weight loss of AlSi37Cu0.7Mg0.9Ni0.2 alloy. (**a**) Single cycle corrosion weight loss; (**b**) total weight loss.

Figure 6 presents the corrosion weight loss behavior of AlSi37Cu0.7Mg0.9Ni0.2 alloy during anodic oxidation under different processing conditions: extruded and solution-treated at various temperatures. The single-cycle corrosion–weight loss curve in Figure 6a showed that the weight loss of all samples remains stable in each cycle, exhibiting an approximately linear trend. Statistical analysis of the 10-cycle cumulative weight loss data (Figure 6b) revealed several critical findings regarding the alloy’s corrosion behavior. The extruded state alloy samples demonstrated optimal corrosion resistance, exhibiting the minimum cumulative mass loss of 19.92 mg·g^−1^. Significantly, the solution-treated alloys displayed a distinct non-monotonic corrosion trend: weight loss initially increased significantly with the rise in solution temperature, followed by a gradual decrease at higher solution temperatures. The solution treatment at 480 °C resulted in peak corrosion susceptibility, with the alloy exhibiting a maximum weight loss of 57.50 mg·g^−1^, representing a 188% increase compared to the extruded condition. Notably, when the solution temperature exceeded 480 °C, a progressive improvement in corrosion resistance was observed. The sample treated at 560 °C demonstrated a reduced weight loss of 27.66 mg·g^−1^, though this value remained 39% higher than that of the extruded alloy samples. The results above demonstrate that solution temperature significantly influences the alloy’s anodic oxidation corrosion behavior, revealing a non-monotonic relationship. The 480 °C treatment represents a critical threshold, corresponding to maximum corrosion susceptibility. Solution treatments above this critical temperature progressively enhance corrosion resistance, suggesting distinct microstructural transformations occur beyond this transition point.

**Figure 7:**
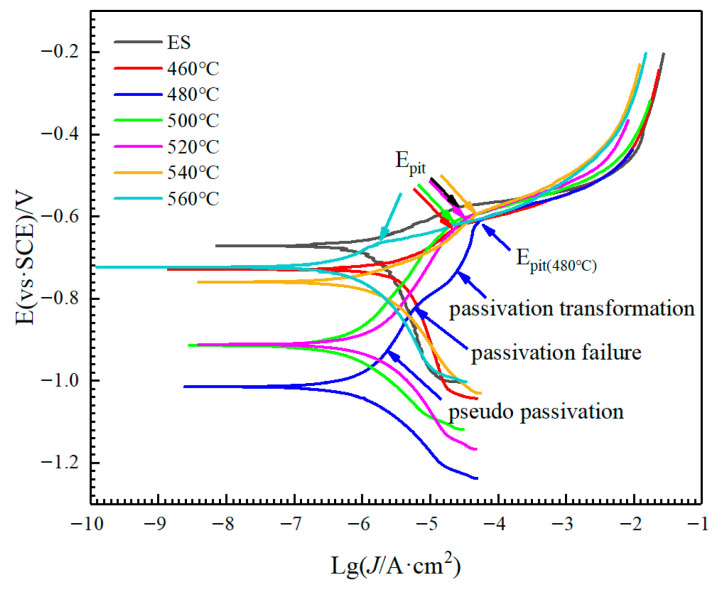
Polarization curves of AlSi37Cu0.7Mg0.9Ni0.2 alloy in 3.5 mass% NaCl solution.

Electrochemical polarization tests were systematically performed to assess the corrosion behavior of AlSi37Cu0.7Mg0.9Ni0.2 alloy under various conditions, as illustrated in Figure 7. According to the Tafel analysis, the corrosion potentials and current corrosion densities of each group of curves can be obtained, and the pitting potentials of the curves were calibrated, as shown in Table 2. The results showed that the corrosion potential followed a descending order: *E_ES_* > *E*_560_ °C > *E*_460_ °C > *E*_540_ °C > *E*_520_ °C > *E*_500_ °C > *E*_480_ °C, where the extruded state alloy showed the highest potential (−0.67244 V) and the 480-treated sample the lowest (−1.0159 V). Conversely, corrosion current densities ranked as *J*_ES_ > *J*_460_ °C > *J*_540_ °C > *J*_520_ °C > *J*_480_ °C > *J*_560_ °C > *J*_500_ °C, with the as-extruded state displaying the highest value. For the extruded state of AlSi37Cu0.7Mg0.9Ni0.2 alloy, as the electrode voltage increased, the corrosion current density increased rapidly for a period of time and then increased slowly, resulting in a certain degree of pseudo passivation and then reaching the *E_pit_* (Pitting Potential). Pitting corrosion occurred on the surface of the alloy at this point, and the passivation film ruptured, followed by rapid dissolution of the alloy. With the increase in solution temperature, the polarization curves of the alloy exhibited a similar change pattern, but the phenomenon of pseudo-passivation became weaker and weaker. As the solution temperature increased to 560 °C, the curve of anodic dissolution was smooth, and there was no obvious pitting tendency. This is because the increase in solution temperature can enhance the dissolution of elements in the matrix. After aging, the phases in the alloy precipitated uniformly, which improved the consistency of the microstructure and reduced the tendency for pitting corrosion. The current density reflects instantaneous localized corrosion rates; corrosion potential serves as a more reliable indicator of overall material susceptibility. At the same time, the level of pitting potential in different states reflects the rupture tendency of the passivation film in the solution, which reflects the corrosion sensitivity of the alloy to some extent. It is worth noting that when the solution temperature was 480 °C, the alloy experienced frequent fluctuations in current during the anodic dissolution process, followed by pseudo-passivation, passivation failure, passivation transformation, and finally pitting corrosion. The rapid change in the alloy surface in the solution under this state reflected the sensitivity of the alloy during anodic dissolution. The combined electrochemical data conclusively demonstrated that 480 °C solution treatment induces the most severe corrosion tendency, which aligns well with the anodizing weight loss measurements and further corroborates its adverse impact on the alloy’s corrosion resistance.

### 3.3. Microstructure

**Figure 8:**
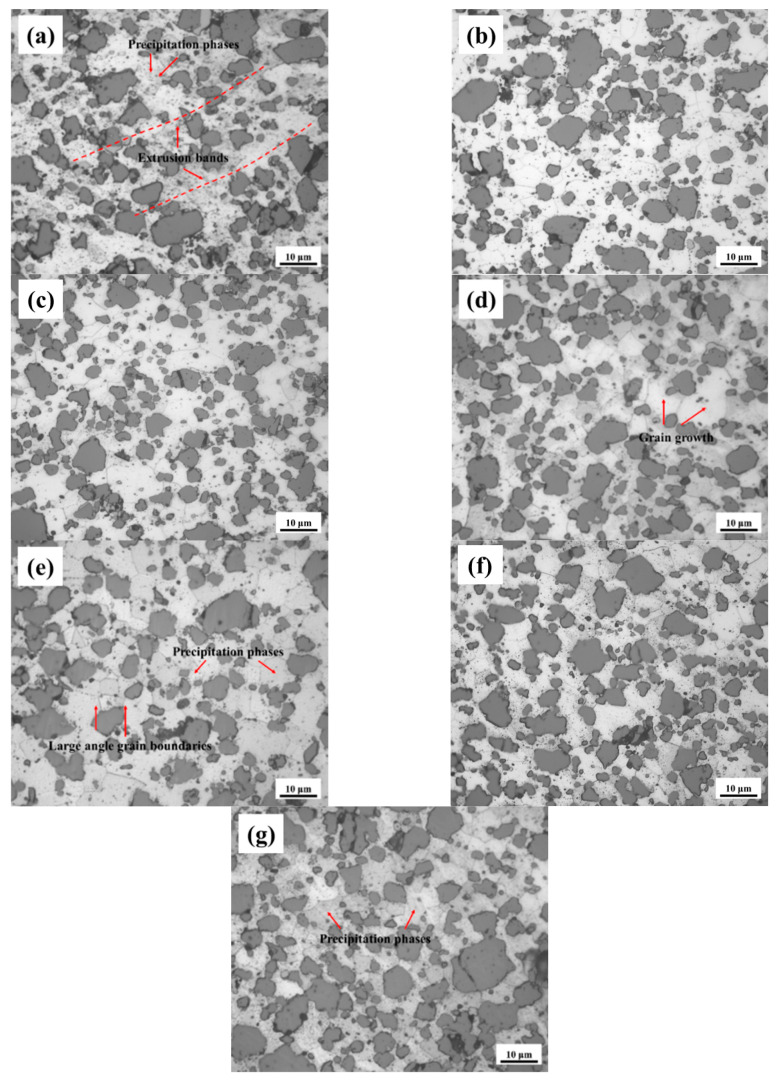
Metallographic microstructures of AlSi37Cu0.7Mg0.9Ni0.2 alloy under different states. (**a**) ES; (**b**) 460 °C; (**c**) 480 °C; (**d**) 500 °C; (**e**) 520 °C; (**f**) 540 °C; (**g**) 560 °C.

The metallographic microstructure of AlSi37Cu0.7Mg0.9Ni0.2 alloy under extruded state and different solution temperatures is shown in Figure 8. It can be seen that the *α*-Al matrix of the extruded alloy exhibited the typical deformation microstructure characterized by obvious extrusion streamlines and blurred grain boundary contours. At the same time, there existed a large number of small precipitates distributed in the *α*-Al matrix. The results demonstrated that the alloy experienced incomplete dynamic recrystallization under the specified extrusion parameters (bar heating temperature: 450–460 °C, extrusion outlet temperature: 380–390 °C) [26,27,28,29]. The microstructure retained substantial processing-induced deformation features, while significant second-phase precipitation occurred during both the extrusion and subsequent cooling stages.

The metallographic microstructures are shown in Figure 8b–g after the solution and aging treatment of the extruded alloy. The results showed that the deformation streamlines of the original extruded state rods gradually disappeared with the increase in solution temperature. In addition, the contours of grain boundaries gradually became clearer and expanded to a certain degree. The graphs indicated that the degree of recrystallization increased as the solution temperature rose. The microstructure exhibited incomplete recrystallization characteristics, with clear grain boundaries observed only in localized areas at the temperature of 460–480 °C; and the recrystallized grains were small and uniform in size (Figure 8b,c). When the solution temperature rose to 500 °C, the recrystallized grains underwent significant coarsening (Figure 8d). It is worth noting that as the temperature continued to rise to 520–560 °C, the microstructure of the alloy exhibited significant evolutions. On the one hand, the grain size magnified further and reached the maximum at 560 °C (Figure 8g). On the other hand, the second-phase particles in the crystals exhibited significant aggregation and coarsening, and their distributions tended to become more uniform.

**Figure 9:**
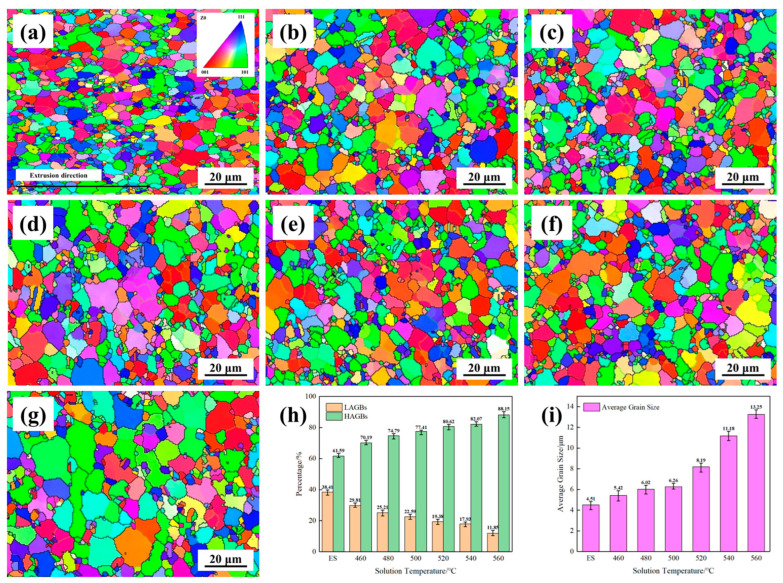
EBSD microstructure of AlSi37Cu0.7Mg0.9Ni0.2 alloy under different states, high- and low-angle grain boundary ratio, and grain size distribution of AlSi37Cu0.7Mg0.9Ni0.2 alloy. (**a**) ES; (**b**) 460 °C; (**c**) 480 °C; (**d**) 500 °C; (**e**) 520 °C; (**f**) 540 °C; (**g**) 560 °C; (**h**) grain boundary ratio; (**i**) grain size distribution.

In order to analyze the recrystallization behavior of AlSi37Cu0.7Mg0.9Ni0.2 alloy at different solution temperatures further, EBSD analysis was performed on the microstructure of the alloy samples. Figure 9a–g show the grain orientation distribution of the extruded AlSi37Cu0.7Mg0.9Ni0.2 alloy at different solution temperatures. The black thick lines in the figures represent high-angle grain boundaries (HAGBs, >15°), and the green thin lines represent low-angle grain boundaries (LAGBs, 2°~15°). It can be seen that a certain degree of dynamic recrystallization occurred on the extruded AlSi37Cu0.7Mg0.9Ni0.2 alloy rods. However, it still retained a certain amount of extruded deformation microstructure, and the grains were elongated along the extrusion direction. The crystal grains were uniformly distributed in an equiaxed ellipsoidal shape after solution treatment. The proportion of high and low angle grain boundaries and the distribution of grain size in the statistical grain structure are shown in Figure 9h,i. It can be seen that LAGBs gradually decreased from 38.41% under the extruded state to 11.85% at 560 °C, with the rise in solution temperature indicating a significant increase in the degree of recrystallization that occurred synchronously. The average crystallite size slowly increased from 4.51 μm to 6.26 μm when the solution temperature was below 500 °C. However, the grain size began to grow significantly as the solution temperature exceeded 520 °C and increased to 13.25 μm at 560 °C. The results above indicated that the degree of recrystallization and the average size of crystal grains of extruded alloy rods gradually increased with the rise in solution temperature. When the solution temperature reached above 520 °C, the crystallite size of the alloy rods increased significantly.

The anodic oxidation of aluminum alloys is the process in which aluminum loses electrons as the anode and combines with oxygen ions in the electrolyte to form the oxide film. The electrolytic reaction can be simply described as follows:

(1) $$H_{2}O\to \left[O\right]+2H^{+}+2e^{-}$$

(2) $$2Al+3\left[O\right]\to {Al}_{2}O_{3}$$

**Figure 10:**
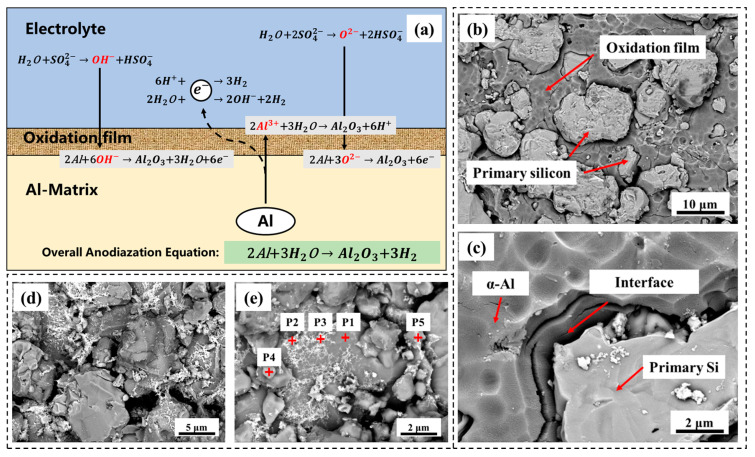
Anodizing reaction mechanism and SEM morphology of anodic oxidation corrosion of AlSi37Cu0.7Mg0.9Ni0.2 alloy. (**a**) Anodizing reaction mechanism; (**b**,**c**) anodized surfaces; (**d**,**e**) corrosion products.

Generally speaking, two competitive reactions occur simultaneously in the anode region during the anodic oxidation of aluminum alloys: one is the electrochemical oxidation reaction of aluminum element (Al → Al^3+^ + 3e^−^), which combines with oxygen to form the aluminum oxide film (2Al^3+^ + 3O^2−^ → Al_2_O_3_); The other is the chemical dissolution reaction of oxide film in electrolyte (Al_2_O_3_ + 6H^+^ → 2Al^3+^ + 3H_2_O) [30,31,32]. The detailed reaction process is shown in Figure 10a. When the generation rate and dissolution rate of the oxide film reached dynamic equilibrium, an anodized film of specific thickness and uniform microstructure can be formed under the specific anodizing process.

Figure 10b,c show the SEM morphology of the surfaces of AlSi37Cu0.7Mg0.9Ni0.2 alloy samples after 10 anodizing cycles. The results displayed that the typical anodic oxide film of porous honeycomb-like was formed on the surface of the *α*-Al matrix after cyclic oxidation corrosion, which gradually dissolved and consumed as the reaction proceeded. However, the oxide film was not formed on the surface of the primary Si particles because of chemical inertness. As the process of anodic oxidation continued, corrosion products deposited on the surfaces of both the *α*-Al matrix and primary Si particles (Figure 10d,e). The results of EDS tests were shown in Table 3. Based on EDS analysis (P2, P3), the corrosion products—predominantly appearing as white net-like structures—were primarily attached to the *α*-Al matrix and consisted mainly of Cu-rich compounds. The phases rich in Cu can reduce electrode potential, and thus, pitting corrosion occurs first nearby. As the α-Al matrix dissolved, the corrosion products remained on the surface. Quantitative analysis indicated that only trace amounts of primary Si particles remained, which were found to coexist with intermetallic compounds containing Cu and Ni (P4, P5). The results demonstrated that the electrochemically active α-Al phase predominantly participated in the anodic oxidation reaction [30,33,34,35], while the more stable alloying elements (Cu, Ni, and Si) remained electrochemically inert and were retained on the matrix surface. The SEM morphology revealed that the primary Si particles obstructed the formation of a complete and dense anodic oxide film on the AlSi37Cu0.7Mg0.9Ni0.2 alloy surface. The bare aluminum metal was continuously exposed at the interface between the *α*-Al matrix and the primary Si particles, forming effective electron transfer pathways that sustained the surface conductivity of the AlSi37Cu0.7Mg0.9Ni0.2 alloy.

**Figure 11:**
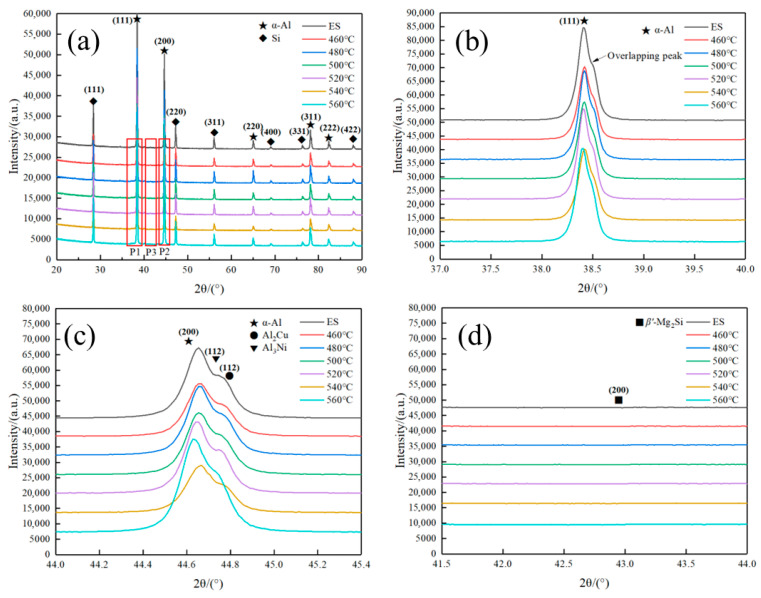
XRD diffraction patterns of AlSi37Cu0.7Mg0.9Ni0.2 alloy under different states. (**a**) 20–90°; (**b**) position 1; (**c**) position 2; (**d**) position 3.

Figure 11 presents the XRD patterns of AlSi37Cu0.7Mg0.9Ni0.2 alloy under both the extruded and solution-treated conditions. The diffraction profile in Figure 10a revealed that the main diffraction peaks of the alloy came from the *α*-Al matrix and the elemental Si phase. The extruded sample demonstrated notable peak broadening at the α-Al (111) reflection (38.5°), as shown in Figure 11b. The broadening of the diffraction peak may be associated with the defects, such as dislocations and stacking faults, formed during hot extrusion or peak overlap with secondary phases. This phenomenon gradually diminished with the increase in solution temperatures, suggesting progressive dissolution of precipitates and strain relaxation in the *α*-Al matrix. As the solution temperature increased, the eutectic Si phase underwent substantial spheroidization. Despite the extremely limited solid solubility of Si in the *α*-Al matrix, complete dissolution occurred during subsequent aging treatment, leading to the precipitation of the elemental Si phase. This transformation process progressively diminished the peak overlapping phenomenon.

The overlapping diffraction peaks observed in the *α*-Al (200) crystal plane region (centered at approximately 44.7°) were systematically analyzed as shown in Figure 11c. Comprehensive phase identification confirmed significant contributions from Al_3_Ni (44.7°, (112)), as well as Al_2_Cu (44.8°, (112)) intermetallic phases [36,37,38]. Due to the low temperature during the extrusion process, these intermetallic compounds were not fully dissolved back into the matrix, resulting in overlap with the *α*-Al (200) peak.

For the *θ*″-Al_2_Cu phase, the increase in solution temperature promoted its progressive dissolution into the *α*-Al matrix, forming a supersaturated solid solution that subsequently transformed into *θ*′-Al_2_Cu precipitates during aging. The phase evolution was evidenced by the gradual attenuation of overlapping diffraction peaks in the 44.7° region. In contrast, the diffusion rate of Ni in the *α*-Al matrix was significantly slower, and the Al_3_Ni phase exhibited remarkable thermal stability. Even after solution treatment at 560 °C, a substantial amount of Al_3_Ni phase remained preserved. Notably, the (200) diffraction peak of the *β*″-Mg_2_Si phase (~42.9°) was not distinctly observed in the XRD pattern due to its relatively weak intensity.

**Figure 12:**
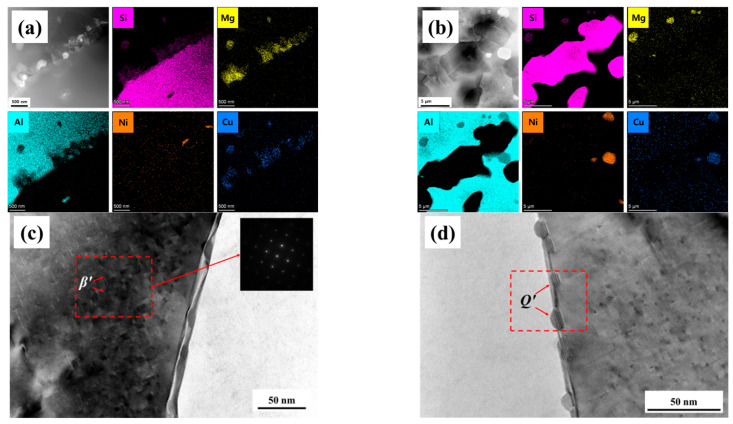
TEM images of AlSi37Cu0.7Mg0.9Ni0.2 Alloy samples. (**a**) Area-scan microstructure of extruded state; (**b**) area-scan microstructure of solution at 540 °C; (**c**,**d**) TEM bright-field images at 540 °C.

Figure 12 presents the TEM analysis results of the extruded state and 540 °C solution-aged AlSi37Cu0.7Mg0.9Ni0.2 alloy samples. The compositional distribution maps revealed that the microstructure of extruded state alloy rods primarily consisted of *α*-Al matrix and primary Si particles, with alloying elements mainly existing in the form of Mg_2_Si phases and thermodynamically stable Al_3_Ni phases. Notably, the distribution of Cu overlapped significantly with that of Mg element, which suggested that a portion of Cu element may exist as the AlCuMgSi quaternary phases. In addition, minor elemental segregation was also observed (as shown in Figure 12a). After solution treatment at 540 °C, most alloying elements dissolved into the *α*-Al matrix to form the supersaturated solid solution, except for insoluble phases such as Al_3_Ni. The strengthening phases, i.e., *β*″-Mg_2_Si, *θ*″-Al_2_Cu, and AlCuNi ternary compounds re-precipitated subsequently during the aging process at 180 °C for 6 h (Figure 12b). The bright-field images of TEM in Figure 12c showed that needle-like *β*″-Mg_2_Si phases (size < 20 nm) were uniformly distributed within the crystals after aging, which maintained coherency with the *α*-Al matrix. In addition, *Q*′ (AlCuMgSi) phases were observed at grain boundaries that were distributed discontinuously (as shown in Figure 12d). The morphology, size, and distribution characteristics of these precipitates altogether determined the mechanical and corrosion properties of the AlSi37Cu0.7Mg0.9Ni0.2 alloy.

For precipitation-strengthened aluminum alloys, the primary objectives of solution treatment are threefold: (1) maximizing solute atom concentration in the matrix to facilitate subsequent precipitation; (2) controlling recrystallized grain growth to maintain optimal mechanical properties; (3) minimizing interfacial weakening caused by the dissolution of low-melting-point non-equilibrium phases. For alloys containing insoluble phases such as Al–Si systems, the influence of Si particle-coarsening on mechanical properties must be carefully considered. While elevated solution temperatures enhance solute atom dissolution into the matrix, excessive temperatures can also cause recrystallization of the alloy, resulting in an increase in grain size and a decrease in the strength and ductility of the alloys. An insufficient solution temperature leads to limited solute atom dissolution in the *α*-Al matrix, which subsequently reduces the population density of strengthening precipitates formed during aging treatment, ultimately compromising the alloy’s strength. Furthermore, the persistence of excessive undissolved secondary phases can promote localized stress concentrations during plastic deformation, thereby deteriorating the alloy’s ductility.

Simultaneously, the solution temperature significantly influences the precipitation behavior of alloy phases by modulating their spatial distribution and morphological characteristics, thereby exerting a profound impact on the corrosion resistance of alloys. Therefore, an optimal solution temperature window must be established to achieve balanced mechanical properties combining high strength with adequate ductility. The above research indicates that for AlSi37Cu0.7Mg0.9Ni0.2 alloy rods prepared by rapid solidification–vacuum hot pressing–hot extrusion deformation, the solution temperature significantly affects the grain structure, alloy phase precipitation behavior, mechanical properties, and anodic oxidation corrosion resistance of the alloy. Consequently, precise temperature control must be implemented according to specific application requirements.

## 4. Conclusions

In this study, AlSi37Cu0.7Mg0.9Ni0.2 alloy rods were fabricated via rapid solidification, followed by vacuum hot pressing and hot extrusion deformation. The influence of solution temperature on microstructural evolution, mechanical properties, and corrosion behavior was systematically investigated. The main results of this study can be summarized as follows:

(1) As the solution temperature increased, the Vickers hardness, yield strength, and tensile strength of AlSi37Cu0.7Mg0.9Ni0.2 alloy gradually increased from 95HV, 109 MPa, and 175 MPa under the extruded state to 198.4HV, 254 MPa, and 282 MPa, respectively, at the solution temperature of 560 °C; the elongation at break gradually decreased from 4.0% to 1.0%. The conductivity of the alloy at the solution temperature of 460 °C (27.44% IACS) was higher than that of the extruded state (26.53% IACS), while the conductivity gradually decreased with the rise in solution temperature.
(2) The extruded state alloy exhibited the best corrosion resistance through anodic oxidation, with a cumulative weight loss of 19.92 mg·g^−1^. As the solution temperature increased, the corrosion weight loss showed a trend of first increasing and then decreasing. The corrosion sensitivity of the alloy reached its maximum value with a cumulative weight loss of 57.50 mg·g^−1^ at 480 °C solution treatment. The self-corrosion potential of the electrochemical polarization curves was consistent with the results of corrosion weight loss.
(3) With the increase in solution temperature, the fraction of low-angle grain boundaries (LAGBs) progressively decreased from 38.41% under the extruded state to 11.85% at 560 °C, accompanied by an increase in average grain size from 4.51 μm to 13.25 μm. Following solution treatment, the majority of alloying elements dissolved into the matrix to form supersaturated solid solutions, with the exception of insoluble phases such as Al_3_Ni. The re-precipitation of *β*″-Mg_2_Si strengthening phases and AlCuNi ternary compounds occurred, along with the formation of discontinuous *Q*′ (AlCuMgSi) phases distributed along grain boundaries during subsequent aging treatment. These precipitation characteristics, including their morphology, size distribution, and spatial arrangement, collectively govern the resultant mechanical properties and corrosion behavior of AlSi37Cu0.7Mg0.9Ni0.2 alloy.

## Figures and Tables

**Table 1:** Chemical composition of AlSi37Cu0.7Mg0.9Ni0.2 alloy (%, mass fraction) [24].

Si	Fe	Cu	Mg	Ni	Mn	Al
36.5–38	0.4	0.65–0.75	0.8–1.0	0.15–0.25	≤0.10	Bal.

**Table 2:** Corrosion potential and current density of AlSi37Cu0.7Mg0.9Ni0.2 alloy in 3.5 mass% NaCl solution.

Number	*E*_corr_/V	*E*_pit_/V	*J*_corr_/(μA·cm^−2^)
ES	−0.67244	−0.57936	0.3303
460	−0.72933	−0.63105	0.20069
480	−1.0159	−0.61579	0.10683
500	−0.91501	−0.62015	0.056329
520	−0.91214	−0.60589	0.13351
540	−0.76026	−0.59874	0.14685
560	−0.72362	−0.66028	0.080878

**Table 3:** Components of EDS tests.

Element	P1	P2	P3	P4	P5
C	2.95	4.80	8.51	13.61	13.43
O	0.57		1.81	9.99	3.09
Mg			2.32	0.95	10.31
Al	92.16	41.21	42.07	5.21	28.27
Si	4.32	43.65	37.54	67.70	36.33
Fe					5.70
Ni					2.88
Cu		10.34	7.75	2.54	
Total	100.00	100.00	100.00	100.00	100.00

## Data Availability

The original contributions presented in this study are included in the article. Further inquiries can be directed to the corresponding authors.
